# Comparing Glaucoma Progression on 24-2 and 10-2 Visual Field Examinations

**DOI:** 10.1371/journal.pone.0127233

**Published:** 2015-05-15

**Authors:** Harsha L. Rao, Viquar U. Begum, Deepa Khadka, Anil K. Mandal, Sirisha Senthil, Chandra S. Garudadri

**Affiliations:** VST Glaucoma Center, L V Prasad Eye Institute, Hyderabad, India; Sun Yat-sen University, CHINA

## Abstract

**Purpose:**

To compare the rate of mean deviation (MD) change on 24-2 versus 10-2 VFs in treated glaucomatous eyes with 5 or more examinations.

**Methods:**

In a retrospective study, 24-2 and 10-2 VFs of 131 glaucoma patients (167 eyes) who had undergone at least 5 VFs examinations during their follow-up were analyzed. All these patients had VF defects both on 24-2 and 10-2 VFs. Rates of MD change were calculated using best linear unbiased predictions (BLUP).

**Results:**

Median age, MD on 24-2 VF at baseline, number of VFs performed during follow-up and follow-up duration were 55 years, -16.9 dB, 9 and 9 years respectively. Median rate of MD change was significantly greater (p<0.001) on 10-2 VF (-0.26 dB/year; interquartile range [IQR]: -0.47, -0.11) compared to 24-2 VFs (-0.19 dB/year; IQR: -0.41, -0.03). Comparing the rates of MD change in eyes with different severities of VF loss (early [MD better than -6 dB], moderate [-6 dB to -12 dB], advanced [-12 to -20 dB] and severe [MD worse than -20 dB]) at baseline (based on the MD on 24-2 VF), median rate of MD change was comparable between 10-2 and 24-2 VFs in mild (-0.45 dB/year vs. -0.40 dB/year, *P* = 0.42) and moderate (-0.32 dB/year vs. -0.40 dB/year, *P* = 0.26) VF loss categories, while the same were significantly greater on 10-2 VFs in advanced (-0.28 dB/year vs. -0.21 dB/year, *P* = 0.04) and severe (-0.18 dB/year vs. -0.06 dB/year, *P*<0.001) VF loss categories.

**Conclusions:**

In patients with VF defects both on 24-2 and 10-2 VFs, evaluating the rate of MD change on 10-2 VFs may help in better estimation of glaucoma progression, especially so in eyes with advanced glaucoma at baseline.

## Introduction

Glaucoma is a progressive optic neuropathy characterized by typical optic disc and retinal nerve fiber layer changes with or without visual field (VF) defects. Automated perimetry is a standard test to evaluate the VF defects in glaucoma. One of the most commonly used automated perimetry is the Humphrey Field analyzer (HFA, Zeiss Humphrey Systems, Dublin, CA). In addition to the standard protocol measuring the central 24 degrees of the VF (24–2 program), HFA also has a protocol to examine the central 10 degrees of the VF with higher resolution (10–2 program). The 24–2 program tests a total of 54 points that are 6 degrees apart and 10–2 program examines 68 test points that are 2 degree apart. The 24–2 program tests only 12 points within the central 10 degrees of fixation. A study by Schiefer et al has shown better detection of VF defects in eyes with suspected glaucoma when closely spaced test points were used.[1] Traynis et al have also reported VF defects on 10–2 VFs in eyes with normal 24–2 VFs.[2]

The management decisions in glaucoma depend not only on the presence of the VF defects but also on their progression. Similar to studies on detecting VF defects, a study by Nevalainen et al has also shown better detection of VF progression with closely spaced test points.[3] In spite of the previous studies showing better detection of VF defects and their progression with closely spaced test points, there is sparse literature on the comparison between commercially available 24–2 and 10–2 VF programs for detecting progression in glaucomatous eyes.[4]

The purpose of this study was to compare the rate of mean deviation (MD) change on 24–2 and 10–2 VFs in glaucomatous eyes with at least 5 examinations.

## Methods

This was a retrospective study of all glaucoma patients who had undergone 5 or more VFs between January 2000 and March 2014 at a tertiary eye care center in India. Written informed consent was obtained from all subjects and the study was approved by the Ethics Committee of L V Prasad Eye Institute. All methods adhered to the tenets of the Declaration of Helsinki for research involving human subjects. One of the four physicians (HLR, AKM, SS, CSG) diagnosed and treated all these patients. Glaucoma was diagnosed based on the structural changes (focal or diffuse neuroretinal rim thinning, localized notching or nerve fiber layer defects) and correlating VF defects.

VF examination was performed using HFA with the Swedish interactive threshold algorithm (SITA) standard 24–2 and 10–2 programs with Goldmann size III stimulus. VF defects were considered glaucomatous if they fulfilled Anderson’s criteria (3 or more non-edged points in a cluster depressed to *P*<5% and 1 of which is depressed to *P* <1%, Glaucoma Hemifield Test outside normal limits and Pattern Standard Deviation depressed to *P* <5%) on 24–2 VF.[5] VFs with fixation losses of > 20% or false positive response rates of >15% were classified as “low reliability”, according to the manufacturer’s recommendation,[6] and excluded from the analysis. Eyes which underwent cataract surgery during the follow-up (either a simple cataract extraction or a combined cataract and glaucoma surgery) were also excluded from the analysis. Eyes with any coexisting retinal or neurological conditions that confounded the results of VF examination were also excluded. All patients had VF defects on 24–2 VF involving at least one of the central 4 cardinal points, the threshold of which was depressed by an amount significant at *P*<5%. A 10–2 VF was done in all these patients, after the 24–2 VF, in the same sitting to evaluate the central VF defect in greater detail.

## Statistical Analyses

Descriptive statistics included mean and standard deviation for normally distributed variables and, median and interquartile range (IQR) for non-normally distributed variables. Shapiro Wilk test was used to test the distribution of data for normality. Rates of MD change on 24–2 and 10–2 VFs were estimated by best linear unbiased predictions (BLUP). BLUPs have been used previously to estimate rates of change in glaucoma.[7–9] Depending on the normality of the distribution, T test or Wilcoxon signed rank test was used to compare the rates of MD change on 24–2 and 10–2 VFs. Statistical analyses were performed using commercial software (Stata ver. 12.1; StataCorp, College Station, TX). A p value of ≤ 0.05 was considered statistically significant.

## Results

We analyzed a total of 182 eyes of 139 glaucoma patients who had VF defects both on 24–2 and 10–2 VFs, and had performed at least 5 VFs during their follow-up, for inclusion into the study. Eight eyes of 4 patients which had undergone cataract surgery during the follow-up were excluded. Further, 77 24–2 VFs and 67 10–2 VFs with “low test reliability” (10 eyes with low reliability on both 24–2 and 10–2 VFs) were excluded, leaving 1355 24–2 and 1355 10–2 VFs from 167 eyes of 131 patients for the final analysis. Median age of the patients at baseline was 55 years (IQR: 46, 65). Glaucoma type consisted of normal tension glaucoma in 19 eyes, primary open angle glaucoma in 86 eyes, primary angle closure glaucoma in 55 eyes and secondary glaucoma in 7 eyes. Median best corrected vision at baseline was 20/25 (IQR: 20/20, 20/30) and spherical refractive error was 0 diopters (-0.5, 1.5). Median MD on 24–2 and 10–2 VF at baseline was -16.9 dB (IQR: -24.3, -10.6) and -14.9 dB (-20.0, -9.8) respectively. Median number of VFs performed was 9 (IQR: 8, 10). Median follow-up duration was 9.0 years (IQR: 7.3, 10.3). Median best corrected vision at last visit was 20/25 (IQR: 20/20, 20/30).

**Fig 1:**
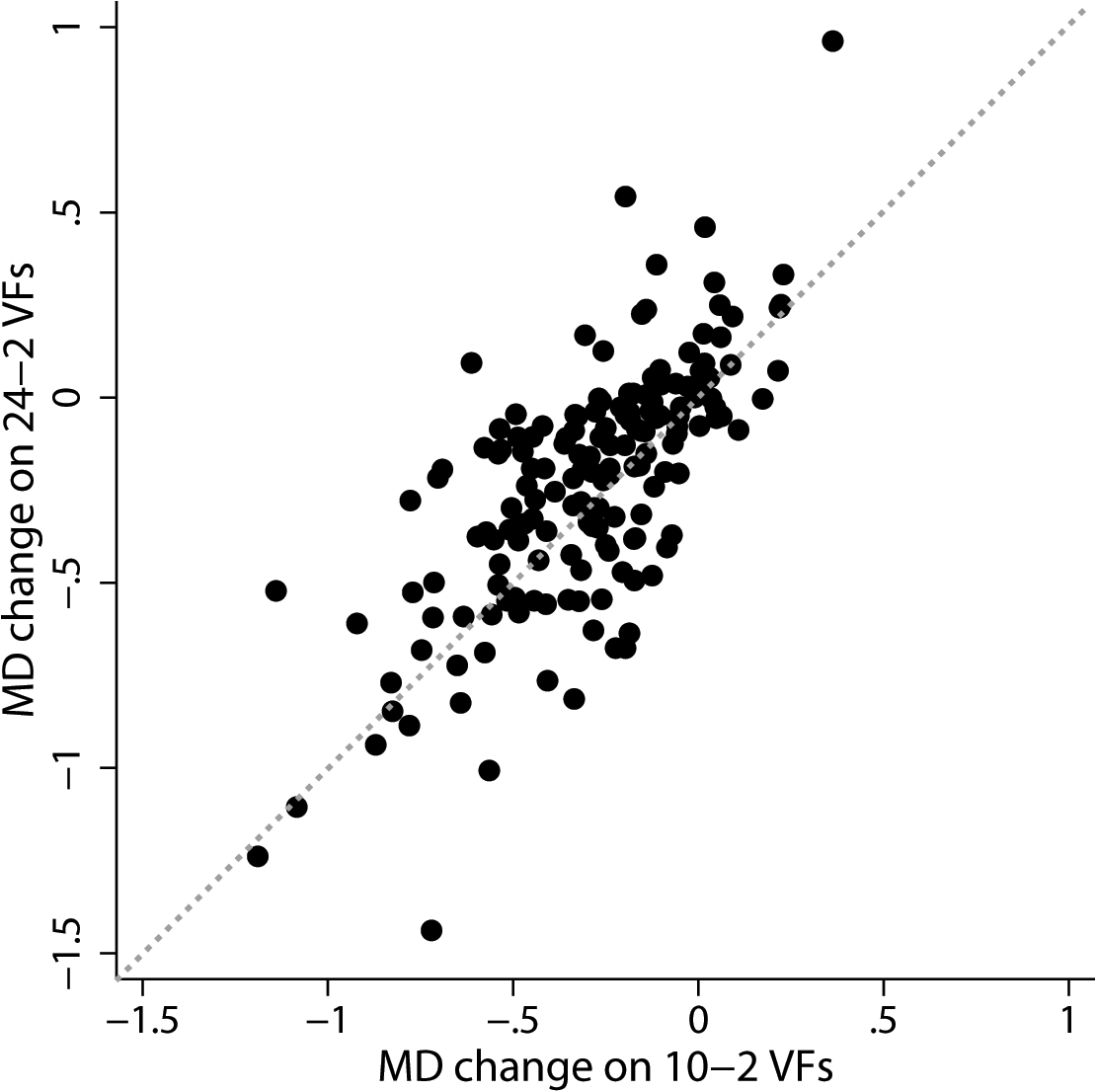
Rate of mean deviation (MD) change on 10–2 and 24–2 visual fields. Scatterplot showing the rate of MD change on 24–2 and 10–2 visual fields (VFs) in individual eyes.

Fig 1 shows a scatter plot demonstrating the MD change on 24–2 and 10–2 VFs in individual eyes. Median rate of MD change was -0.19 dB/year (IQR: -0.41, -0.03) on 24–2 and -0.26 dB/year (IQR: -0.47, -0.11) on 10–2 VFs. MD change on 10–2 VFs was significantly greater than that on 24–2 VFs (p<0.001). Median difference in rate of MD change between 10–2 and 24–2 VFs (MD change on 10–2 VF minus MD change on 24–2 VF) was -0.06 dB/year (IQR: -0.19, +0.07).

Table 1 shows the effect of age at baseline and severity of VF loss at baseline on the rate of MD change on 24–2 and 10–2 VFs. Rates of MD change on both 24–2 and 10–2 VFs were greater (more negative) in older patients and were lesser (less negative) in eyes with more severe VF loss at baseline.

**Table 1:** Effect of baseline age and severity of visual field (VF) loss at baseline (based on mean deviation [MD] on 24–2 VF) on the rates of MD change on 24–2 and 10–2 VFs.

	Effect on MD change on 24–2 VF		Effect on MD change on 10–2 VF	
	Coefficient (95% CI)	P value	Coefficient (95% CI)	P value
Baseline age	-0.01 (-0.01, -0.002)	0.002	-0.004 (-0.01, -0.001)	0.005
Baseline MD	-0.02 (-0.02, -0.01)	<0.001	-0.01 (-0.01, -0.01)	<0.001

**Fig 2:**
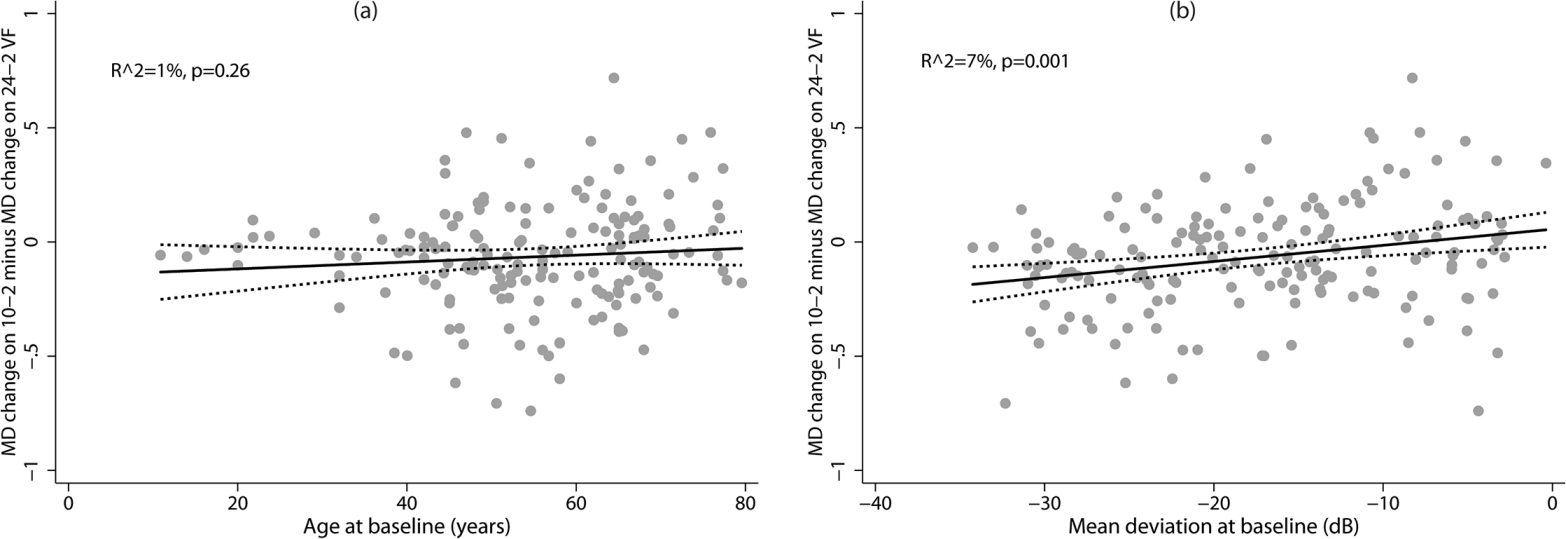
Influence of age and severity of glaucoma on the rate of mean deviation (MD) change on 10–2 and 24–2 visual fields. Difference in the rate of MD change between 10–2 and 24–2 fields and its relationship with (2a) age at baseline and (2b) severity of visual field loss (MD on 24–2 field) at baseline.

Fig 2 shows the effect of age at baseline and VF severity at baseline (MD on 24–2 VF) on the difference in rate of MD change between 10–2 and 24–2 VFs. Baseline age did not have an association with the difference in the rate of MD change between 10–2 and 24–2 VFs. Baseline glaucoma severity had a statistically significant association with the difference in rate of MD change between 10–2 and 24–2 VFs with the rate of MD change on 10–2 VFs being significantly greater than that on 24–2 VFs in eyes with more severe glaucomatous VF loss at baseline. To evaluate this result further, we divided the study group based on the baseline glaucoma severity into mild (MD better than -6 dB), moderate (MD between -6.01 and -12 dB), advanced (MD between -12.01 and -20 dB) and severe (MD worse than -20.01 dB) VF loss categories. Table 2 shows the characteristics of these categories. Table 3 shows the rate of MD change on 10–2 and 24–2 VFs in mild, moderate, advanced and severe VF loss categories at baseline. Rate of MD change was similar on 24–2 and 10–2 VFs in eyes with mild and moderate VF loss at baseline while in eyes with advanced and severe VF loss at baseline, rate of MD change was significantly greater on 10–2 compared to 24–2 VFs.

**Table 2:** Characteristics of the eyes with mild (mean deviation [MD] better than -6 dB), moderate (MD between -6.01 and -12 dB), advanced (MD between -12.01 and -20 dB) and severe (MD worse than -20.01 dB) VF loss at baseline.

	Mild glaucoma (24 eyes)	Moderate glaucoma (27 eyes)	Advanced glaucoma (48 eyes)	Severe glaucoma (68 eyes)	P value
Age (years)	54 (48, 64)	60 (47, 65)	56 (48, 67)	54 (45, 65)	0.45
Glaucoma type (n)	NTG: 1POAG: 11PACG: 10	NTG: 3POAG: 16PACG: 7	NTG: 8POAG: 25PACG: 13	NTG: 7POAG: 34PACG: 25	0.59
Follow-up (years)	8.5 (6.3, 10.4)	8.3 (7.3, 10.2)	9.1 (7.6, 10.3)	9.3 (7.7, 10.5)	0.70

NTG: normal tension glaucoma; POAG: primary open angle glaucoma; PACG: primary angle closure glaucoma.

**Table 3:** Median rate of mean deviation (MD) change (interquartile ranges in brackets) in different severities of glaucoma on 24–2 and 10–2 visual fields (VF) at baseline.

Severity of glaucoma	MD change on 24–2 VF	MD change on 10–2 VF	P value
MD>-6 dB (n = 24)	-0.40 (-0.56, -0.18)	-0.45 (-0.55,-0.28)	0.42
MD between -6 & -12 dB (n = 27)	-0.40 (-0.68, -0.18)	-0.32(-0.47, -0.17)	0.26
MD between -12 & -20 dB (n = 48)	-0.21 (-0.46, -0.06)	-0.28 (-0.51, -0.14)	0.04
MD<-20 dB (n = 68)	-0.06 (-0.16, 0.05)	-0.18 (-0.32, -0.05)	<0.001

We also evaluated a binary classification of progression on 24–2 and 10–2 VFs by defining any rate of MD change of < -0.5 dB/year with a p value significant at < 5% as “progression”. Table 4 shows the number of eyes progression by this criteria according to the severity of VF loss at baseline. While the number of eyes progressing on 24–2 VFs was significantly more in early and moderate glaucoma (p<0.001, Chi square test), number of eyes progressing on 10–2 VFs was significantly more in advanced and severe glaucoma (p<0.001, Chi square test).

**Table 4:** Number of eyes progressing on 24–2 and 10–2 visual fields (VF) according to the severity of VF loss at baseline.

	MD > -12 dB at baseline		MD < -12 dB at baseline	
	Progression on 10–2 VF		Progression on 10–2 VF	
	Present	Absent	Present	Absent
Progression on 24–2 VFs	12	7	8	6
No progression on 24–2 VFs	3	29	12	90

Progression was defined as a MD change of <-0.5 dB/year with a p value of <5%. MD: mean deviation.

## Discussion

In this study, we evaluated the rate of MD change in glaucomatous eyes with VF defects on 24–2 and 10–2 VFs. We found that the rate of MD change was similar on 24–2 and 10–2 VFs in eyes with early and moderate glaucomatous VF loss while in eyes with advanced and severe glaucoma, rate of MD change was significantly greater on 10–2 compared to 24–2 VFs.

Though earlier studies have shown the importance of closely spaced test points on VF examination both for detection of VF defects and their progression,[1–3] studies evaluating the rate of glaucoma progression on 24–2 versus 10–2 VFs are limited. Park et al. compared the progression of parafoveal scotoma (MD on 24–2 VF at baseline ranging between -14.1 and 0.2 dB) on 24–2 and 10–2 VFs in 50 glaucomatous eyes.[4] Progression in their study was defined as one or more points with a threshold decrease at the rate of 1 dB/year with *P*<1%. They found significantly more progressing eyes on 10–2 than on 24–2 VF analyses (24 eyes vs. 11 eyes; *P* = 0.007). They also found a significantly greater (*P* = 0.015) rate of MD change on 10–2 VFs (-0.40±0.51 dB/year) compared to 24–2 VFs (-0.23±0.28 dB/year).[4] Our results are slightly different from that found by Park et al. We found comparable rates of MD change on 24–2 and 10–2 VFs in eyes with early and moderate glaucoma on trend-based analysis while the number of eyes classified as progressed based on the binary classification system (though our definition of progression was different from that used by Park et al) was significantly more on 24–2 compared to 10–2 VFs. This difference in results between our study and the study by Park et al may be related to the differences in the characteristics of the included eyes. We included all eyes where the VF defect on 24–2 VF involved one of the 4 central points with a correlating affection on 10–2 VF. Park et al included only eyes with a strictly defined parafoveal scotoma with no points outside the central 10 degrees involved on 24–2 VF.

In eyes with advanced and severe VF loss at baseline, we found that the rate of MD change on 10–2 VF was significantly greater than that on 24–2 VFs. The number of eyes progressing by a binary classification system was also significantly more on 10–2 compared to 24–2 VFs in eyes with advanced and severe VF loss at baseline. This is because of the fact that most of the peripheral points are likely to have reached their “floor” in these eyes with advanced and severe VF loss. Inability to detect further change in these peripheral points would mask the change happening in the central points when the MD is estimated by averaging the sensitivity loss across all points on a 24–2 VF. With only a central island of field remaining in these eyes, it is possible that the change would stand out better when the central VF is evaluated with greater resolution.

We also found that the rate of MD change both on 24–2 and 10–2 VFs was dependent on the age of the patient. Age of the patient has been reported to be associated with VF progression in multiple previous studies, with elder patients showing a greater rate of progression.[10–14] Rate of MD change decreased with increasing severity of VF loss at baseline. This is once again likely related to the “floor” effect discussed above. We have in a previous study evaluated this relationship in greater detail.[15]

We used BLUPs for estimation of rates of MD change in our study. Previous studies have predominantly used the traditional ordinary least squares (OLS) or simple linear regression of the measurements obtained over time, to estimate MD change or threshold change at each tested point of VF (point-wise linear regression analysis).[4,14,16–20] The advantage of BLUPs over the OLS estimates is that the BLUPs are shrinkage estimates that take into account the results obtained by evaluating the whole sample of eyes, while the OLS estimates do not take the entire population into account.[21] Also, BLUPs give less weight to estimates obtained from eyes with few measurements and/or large intra-individual variability and so can be more precise than OLS estimates in such situations.[22] A previous study by Medeiros et al. comparing the BLUP and OLS estimates has also reported significantly better prediction of rates of VF loss with BLUP compared to OLS estimates in glaucoma.[9] One of the concerns here is that the BLUPs are shrinkage estimates and they tend to neglect the outliers.[21] These outliers can be important in glaucoma as they may represent fast progressors. This concern however is unlikely to be significant in our study of comparing the BLUP estimates with two VF testing protocols.

Though MD is a widely used global index to quantify severity of VF loss, it has limitations. MD is affected by media opacities and by other causes of generalized depression of visual function in addition to glaucoma.[23–25] We therefore excluded all eyes that underwent a cataract surgery during the follow-up as the effect of visually significant cataract can be different on the MD of a 24–2 and a 10–2 VF. Visual field index (VFI) is another global measure used to quantify VF loss and progression. VFI has been reported to be less affected by media opacities unlike MD.[26,27] However, VFI estimation is currently available only for 24–2 VFs but not for 10–2 VFs. Pattern standard deviation (PSD) is another global measure that can be used to quantify VF loss. PSD has the advantage that it is less affected by media opacities but has the disadvantage that it improves in eyes with MD worse than -20 dB.[26]

Our study has a few limitations. Rate of MD change is affected by cataract progression as well as IOP fluctuations. Though we did not evaluate the cataract progression in our study, best corrected vision of the eyes at baseline and last visit was similar. We also did not evaluate the IOP fluctuations or the anti-glaucoma treatments in our study. Though both these factors affect the rate of MD change, it is unlikely that the rates of MD change on 24–2 and 10–2 VFs are affected differently. However, further studies are needed to conclusively address these issues. Both the 24–2 and 10–2 VF examinations in the subjects were performed in quick succession in our study. Previous studies have shown the effect of fatigue when VFs are performed without adequate time gap in between.[28–30] Fatigue is known to result in decreased visual sensitivity which is more pronounced at the margins of the VF defect and at more eccentric VF locations. The other limitation of our study is that we only evaluated the change in MD on 24–2 and 10–2 VFs. MD is a global index and focal changes in the VF may get masked when MD alone is considered. Pointwise linear regression analysis of threshold sensitivities at individual points as performed by Park et al[4] as well as isopter evaluations using semi-automated kinetic perimetry[31] could have provided additional information especially in the context of advanced glaucoma.

Our study highlights the importance of following up glaucoma patients, especially those with advanced VF loss, with 10–2 VFs when the central points on 24–2 VFs are involved. With 24–2 VFs, there are commercially available trend-based and event-based progression analysis algorithms (Guided Progression Analysis, Carl Zeiss Meditec, Inc. Dublin, CA), which help the clinicians in decision making. Unfortunately, no such progression analysis algorithms are available for 10–2 VFs. Future research should be directed towards developing similar trend and event-based glaucoma progression analysis algorithms for 10–2 VFs.

In conclusion, our results demonstrate that in patients with VF defects both on 24–2 and 10–2 VFs, evaluating the rate of MD change on 10–2 VFs may help in better estimation of glaucoma progression, especially so in eyes with advanced glaucoma.
